# Exploring the Glycans of *Euglena gracilis*

**DOI:** 10.3390/biology6040045

**Published:** 2017-12-15

**Authors:** Ellis C. O’Neill, Sakonwan Kuhaudomlarp, Martin Rejzek, Jonatan U. Fangel, Kathirvel Alagesan, Daniel Kolarich, William G. T. Willats, Robert A. Field

**Affiliations:** 1 Department of Plant Sciences, University of Oxford, South Parks Road, Oxford OX1 3RB, UK; 2 Department of Biological Chemistry, John Innes Centre, Norwich Research Park, Norwich NR4 7UH, UK; Sakonwan.Kuhaudomlarp@jic.ac.uk (S.K.); Martin.Rejzek@jic.ac.uk (M.R.); 3 Department of Plant and Environmental Sciences, University of Copenhagen, Frederiksberg 1871, Denmark; Jonatan.Ulrik.Fangel@carlsberg.com; 4 Institute for Glycomics, Gold Coast Campus, Griffith University, Southport, QLD 4222, Australia; k.alagesan@griffith.edu.au (K.A.); d.kolarich@griffith.edu.au (D.K.); 5 Department of Biomolecular Sciences, Max Planck Institute of Colloids and Interfaces, 14424 Potsdam, Germany; 6 School of Natural and Environmental Sciences, Newcastle University, Newcastle upon Tyne NE1 7RU, UK; William.Willats@newcastle.ac.uk

**Keywords:** algae, Euglena, biotechnology, carbohydrates, *N*-glycan, sugar nucleotide

## Abstract

*Euglena gracilis* is an alga of great biotechnological interest and extensive metabolic capacity, able to make high levels of bioactive compounds, such as polyunsaturated fatty acids, vitamins and β-glucan. Previous work has shown that Euglena expresses a wide range of carbohydrate-active enzymes, suggesting an unexpectedly high capacity for the synthesis of complex carbohydrates for a single-celled organism. Here, we present an analysis of some of the carbohydrates synthesised by *Euglena gracilis*. Analysis of the sugar nucleotide pool showed that there are the substrates necessary for synthesis of complex polysaccharides, including the unusual sugar galactofuranose. Lectin- and antibody-based profiling of whole cells and extracted carbohydrates revealed a complex galactan, xylan and aminosugar based surface. Protein *N*-glycan profiling, however, indicated that just simple high mannose-type glycans are present and that they are partially modified with putative aminoethylphosphonate moieties. Together, these data indicate that Euglena possesses a complex glycan surface, unrelated to plant cell walls, while its protein glycosylation is simple. Taken together, these findings suggest that *Euglena gracilis* may lend itself to the production of pharmaceutical glycoproteins.

## 1. Introduction

Euglena are a group of fast-growing, mainly freshwater algae, that are distantly related to other algae. They have long been studied for both their fundamental biology and for their production of a range of high value products, including vitamins, amino acids and polyunsaturated fatty acids [1]. As they are easy to grow and have a large metabolic capacity, Euglena have been proposed as a platform for the production of proteins and high value metabolites of biotechnological interest [2] and for the synthesis of complex natural products [3].

The genome sequence of Euglena is not yet available, but *de novo* transcriptome sequencing of the most heavily studied species, *Euglena gracilis*, has unveiled the huge metabolic capacity of these organisms. In fact, Euglena possesses more genes than are evident in the human genome [4]. Of particular note is the large number of carbohydrate-active enzymes (CAZymes), comparable in number to multicellular animals, and much higher than other single-celled algae. However, predicting the specificity of CAZymes is notoriously difficult and it is not currently possible to predict the structure of the carbohydrates made from enzyme sequences alone.

Euglena is not reported to have a carbohydrate-based cell wall, but glucose, galactose, mannose, fucose, xylose, and rhamnose have been detected in cell surface extracts by paper chromatography [5]. It has also been reported that Euglena cells can become encased in a carbohydrate sheath, forming cysts [6]. Euglena has a full complement of genes for the biosynthesis of glycosylphosphatidylinositol (GPI) membrane anchors which may be involved in anchoring these glycans to internal membranes or the cell surface [4]. *N*-Acetylglucosamine-1-phosphate transferase activity has been detected in membrane preparations of Euglena cells [7] and a complex xylose-containing material has been found associated with the flagella [8]. A wide range of glycosyltransferases are present in the Euglena transcriptome, supporting the capacity for the synthesis of complex glycans, although the exact structure of such glycans remains to be elucidated.

Protein glycosylation appears to be fairly typical in Euglena, synthesising the same *N*-glycan precursor oligosaccharide as animals and fungi (Glc_3_Man_9_GlcNAc_2_-Asn) [9], with sequences for all of the enzymes required for the synthesis present in the transcriptome [4]. There are three sequences for the oligosaccharyltransferases that couple this pre-formed oligosaccharide to proteins, in common with *Trypanosomes* where the enzymes have different substrate specificities [10]. In Euglena, flagella-associated glycoproteins are affected by tunicamycin, an inhibitor of protein glycosylation [11]. However, the exact structure of the glycans and the identity of glycosylated proteins remain elusive. There are also three members of GT41 family of protein glycosyltransferases present in the transcriptome [12], more than in animals, which have one protein *O*-GlcNAc transferase [13] or plants, which have both an *O*-GlcNAc transferase and an *O*-fucosyltransferase in this family [14].

The range of tantalising previous work and the rather high number of glycosyltransferases led us to speculate that Euglena has great capacity for the synthesis of complex glycans and we were therefore minded to investigate the carbohydrates synthesised by these organisms.

## 2. Materials and Methods

### 2.1. Growth of Euglena

*Euglena gracilis* var. *saccharophila* Klebs (strain 1224/7a) was obtained from the Culture Collection of Algae and Protozoa (CCAP) (http://www.ccap.ac.uk/) and treated with antibiotics [12]. For heterotrophic cultures, cells were grown in the dark in Euglena gracilis:Jaworski’s (EG:JM) medium (https://www.ccap.ac.uk/media/documents/EG_JM.pdf) containing additional glucose (15 g/L) at 30 °C. For photoheterotrophic cultures, cells were grown in the same media at 22 °C on a 14:10 light cycle with a light intensity of 100 µmol·m^−2^·s^−1^. For photoautotrophic cultures, cells were grown in JM media in the same growth conditions. All cultures were shaken at 150 rpm. 

### 2.2. Lectin Labelling

Heterotrophic cells were collected at mid log phase, resuspended in the recommended lectin buffers and incubated with the appropriate lectins (Vector labs, Burlingame, CA, USA). For aggregation, cells were then visualised after 1 h. For the fluorescently labelled samples, cells were washed with fresh buffer and then visualised using a Leica DM 6000 (Leica microsystems, Milton Keynes, UK) equipped with a DFC420 camera (Leica microsystems, Milton Keynes, UK). Excitations of 450–490 nm and 515–560 nm were used for visualising the fluorescent lectins (Vector labs, Burlingame, CA, USA).

### 2.3. Sugar Nucleotide Profiling

The methods used were essentially as reported by Rejzek et al. [15] and Wagstaff et al. [16]. In brief, mid-log phase (OD_600_ = 1.1 in about 6 days) cultures were harvested (3 biological replicates). UDP-α-d-GlcNAcA was added to the cell pellet as internal standard (1.46 nmol/g wet pellet). Cell lysis was performed with cold (−20 °C) 70% ethanol (20 mL). The cell debris was removed by centrifugation (28,928× *g*, 20 min, 4 °C) and the supernatant was concentrated to dryness. Lipophilic components were removed by partitioning the sample between water and butan-1-ol [17]. Solid phase extraction (SPE) on a graphitised carbon column (EnviCarb, 250 mg, 3 mL Supelco, Bellefonte, PA, USA) was performed essentially as described by Rabina and co-workers [18]. LC-MS/MS profiling of sugar nucleotides was performed on a Xevo TQ-S tandem quadrupole mass spectrometer (Waters) operated in multiple reaction monitoring (MRM) mode coupled to an Acquity UPLC. MRM transitions for sugar nucleotide standards in negative ESI mode were generated using IntelliStart software (Table S2, Supplementary Materials). Samples (10 µM) were introduced at 10 µL/min combined with a flow from the HPLC pump typical of an LC run. MassLynx software (Waters) was used to collect, to analyse and to process data. Separation of sugar nucleotides was achieved on a surface-conditioned PGC column (Hypercarb, Fisher Scientific, Loughborough, UK, dimensions 1 × 100 mm, particle size 5 µm) equipped with a column guard (Hypercarb, 5 µm, 1 × 10 mm) [19]. Analytes were eluted using mobile phase A: formic acid 0.3% brought to pH 9.0 with ammonia and mobile phase B: acetonitrile using the following multistep gradient at a flow rate 80 µL/min: 0 min: 2% B; 20 min: 15% B; 26 min: 50% B; 27 min: 90% B; 30 min: 90% B; 31 min: 2% B; 50 min: 2% B. Available sugar nucleotide standards (10 µM) were injected (5 µL) to determine retention time (Table S2, Supplementary Materials). UDP-α-d-Glc, UDP-α-d-GlcNAc, UDP-α-d-GlcA, UDP-α-d-Gal, TDP-α-d-Glc, ADP-α-d-Glc, ADP-d-Rib, GDP-α-d-Man and GDP-β-l-Fuc were obtained commercially from Sigma Aldrich (Haverhill, UK). UDP-α-d-GlcNAcA [20], TDP-β-l-Rha [16], UDP-β-l-Rha [16], UDP-α-d-Xyl [21] and UDP-β-l-Arap [22] were prepared as previously described. Although between runs there were significant differences in absolute retention times of standards, relative retentions were reasonably reproducible (Table S2, Supplementary Materials). Using a serial dilution of UDP-α-d-Glc limit of detection was determined to be 10 fmol on column. Where in doubt, co-injection of sample with appropriate standard sugar nucleotide was used for positive identification.

### 2.4. Immunocarbohydrate Microarray Profiling

The samples were snap frozen in liquid nitrogen and ground with steel balls in a tissue lyser (Qiagen, Hilden, Germany) for 1 min at 30 s^−1^ frequency. AIR was isolated by washing the homogenate with 70% ethanol followed by drying. Sequential extraction was carried out as described in Moller et al. [23] Briefly, 300 μL of CDTA was added to 10.0 mg of material, and the microtubes were shaken at RT for 2 h. The samples were centrifuged at 4000× *g* for 10 min, and the supernatant was removed and pipetted to a new microtube. NaOH at 4 M + 0.1% (*w*/*v*) NaBH_4_ was added to the sediment and shaken for 2 h. The samples were centrifuged at 4000 *g* for 10 min, and the supernatant was removed and pipetted to new microtubes. The printing on the nitrocellulose membrane was done on an ArrayJet Sprint printer in two replicates and four dilutions, probed, and quantified as described by Moller et al. [24].

### 2.5. N- and O-Glycan Analysis

#### 2.5.1. Sample Preparation

Euglena cells were grown in EG:JM+Glc media in the dark and the flagella isolated by cooling to 4 °C for 1 h prior to centrifugation at 800× *g* to remove the cells and at 20,000× *g* to collect the flagella. About 5 μg of the obtained flagellar protein purified from *Euglena gracilis* was dot-blotted on to the PVDF membrane. *N*- and *O*-glycans were released from the dot-blotted protein as described by Jensen et al. [25]. Briefly, The *N*-linked glycans were released by incubation with PNGase F (3 U) overnight at 37 °C. The released *N*-linked glycans were isolated, dried and reduced with 20 μL of 1 M NaBH_4_ in 50 mM KOH at 50 °C for 3 h. The reduction was quenched with 1 μL glacial acetic acid and *N*-linked glycans were purified using cation exchange columns made from 30 μL AG50W-X8 cation-exchange resin (BioRad, Hercules, CA, USA) packed on μC18 ZipTips. The residual borate was removed by addition of methanol (200 μL) and dried under vacuum. *O*-linked glycans were then released from the *N*-glycan liberated sample by reductive β-elimination performed overnight in 20 μL of 0.5 M NaBH_4_ dissolved in 50 mM KOH at 50 °C. The reduction was quenched with 1 μL glacial acetic acid and *O*-linked glycans were desalted using the cation exchange columns as described above for the *N*-glycans. The purified glycans were then further purified by PGC-SPE and vacuum dried prior PGC LC ESI MS/MS.

#### 2.5.2. Mass Spectrometric Analysis

PGC-LC was performed on an Ultimate 3000 UHPLC system (Dionex, Part of Thermo Fisher, Bremen, Germany) online coupled to an amaZon speed ETD ion trap mass spectrometer (Bruker Daltonics, Bremen, Germany). Dried samples (*N*- and *O*-glycans) were dissolved in 6 μL water and 5 μL were used for separation by PGC-LC (Hypercarb, 30 × 0.32 mm, 5 μm particle size (pre-column), 100 mm × 180 μm, 3 μm particle size (separation column), Thermo Hypersil, Runcorn UK). The sample was loaded on the pre-column at a flow rate of 6 μL/min in buffer A (10 mM ammonium bicarbonate) for 5 min before a gradient of 5.8% to 30.3% buffer B (60% ACN in 10 mM ammonium bicarbonate) was applied over 54 min at a flow-rate of 1 μL/min. The column oven temperature was set to 35 °C. The LC system was connected to the mass spectrometer using the nano-flow ESI sprayer (Bruker Daltonics, Bremen, Germany). The MS settings were as follows: capillary outlet 1100 V. Negative ion mode MS spectra were obtained within a mass range of 380–1800 *m*/*z*. Smart parameter setting (SPS) was set to 900 *m*/*z*. For MS/MS precursor selection, the three most intense ions, including singly charged ones, above the absolute intensity of 34.000 and 20% relative intensity threshold were isolated with a width of 1.0 *m*/*z*. The ICC was set to 40.000 with a maximum accumulation time of 200 ms. CID was performed using helium as collision gas. The fragmentation amplitude was set to 100% using in addition SmartFragTM Enhanced for amplitude ramping (30–120%). Fragmentation time was set to 32 ms. The instrument was controlled using Hystar 3.2 software. MS and MS/MS data were processed using DataAnalysis 4.1 (Bruker, Bremen, Germany). The glycan peaks were semi-quantified using the extracted ion chromatogram peak areas.

## 3. Results and Discussion

### 3.1. Carbohydrate Active Enzymes in Euglena

**Figure 1:**
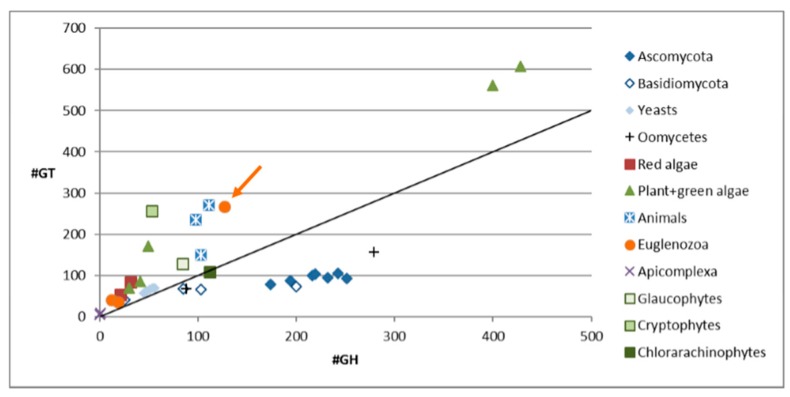
The number of carbohydrate active enzymes annotated in the genomes of selected organisms. Most organisms have more glycosyltransferases than glycoside hydrolases, except amongst the saprophytic fungi and oomycetes. *Euglena gracilis* is indicated with an arrow. See Table S1, Supplementary Materials for a breakdown of the CAZyme families encoded in the Euglena transcriptome.

Inspection of the transcriptome of Euglena [4] showed that there are more CAZymes than in most other sequenced algae, but fewer than in the land plants, which require complex cell walls to support their growth (see Figure 1). There are also many more CAZymes encoded than in the other sequenced Euglenozoa, such as the human pathogens *Trypanosoma brucei* and *Leishmania braziliensis* (orange circles in the lower left). One major change is the loss of enzymes involved in α-glucan biosynthesis and an increase in those involved in synthesis of β-glucans [4]. This reflects the use of paramylon, an insoluble β-1,3-glucan [26], as the storage polysaccharide, as opposed to the α-1,4/6-linked glucan used as the energy store in plants, animals and bacteria (starch or glycogen, respectively). Most β-glucans found in nature are either branched or mixed linkage, such as the 1,3 and 1,6 linkages in fungal cell walls and the storage polysaccharides of sea weeds, or 1,3 and 1,4 linkages found in cereals, such as oat [27]. In contrast, the pure 1,3 linkage of the Euglena paramylon makes it an ideal model system to study the synthesis of this type of carbohydrate.

### 3.2. Sugar Nucleotide Profiling in Euglena

**Figure 2:**
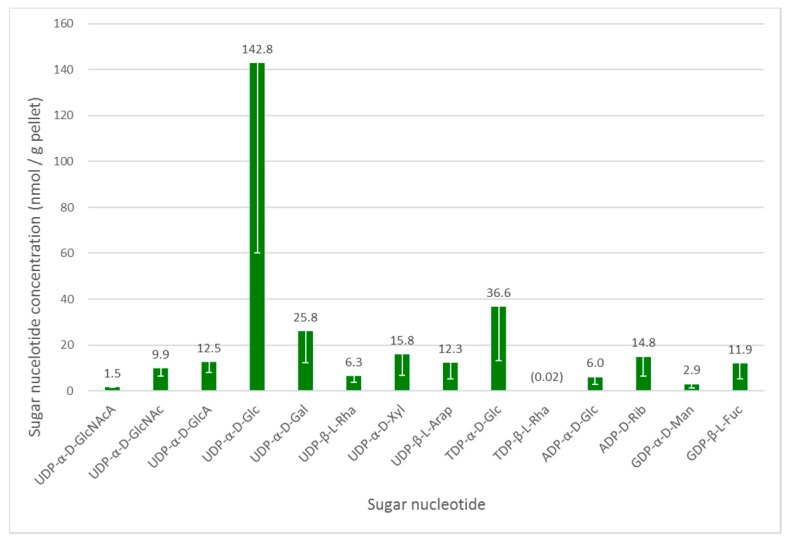
Intracellular sugar nucleotide profile of *Euglena gracilis* grown under photoheterotrophic conditions. An internal standard UDP-α-d-GlcNAcA (1.46 nmol/g pellet) was added to the samples. The data are mean of three biological replicates; error bars (negative value only) indicate standard error. The identity of all NDP-sugar species was confirmed using authentic standards.

As in other organisms, the glycosylation machinery in Euglena depends on the availability of activated sugar building blocks, namely the sugar nucleotides [28]. Euglena cell cultures grown under photoheterotrophic conditions—the intermediate growth condition and likely to give the widest range of nucleotides to serve as a baseline for wider-ranging studies—were analysed to determine the range of these substrates available. To ensure reproducibility, mid-log phase cultures were harvested and an internal standard was added at this stage to enable quantification. UDP-α-d-GlcNAcA was selected as internal standard, based on the fact that it was absent in the Euglena extracts and that it did not co-elute (see Table S2, Supplementary Materials) with any significant analytes. The intracellular sugar nucleotides were extracted by cell lysis with cold 70% ethanol, followed by the removal of lipophilic components and solid phase extraction on a graphitised non-porous carbon column, as described previously [18]. Liquid chromatography separation of sugar nucleotides was achieved on a surface-conditioned porous graphitic carbon (PGC) column using a gradient of acetonitrile against 0.3% (*v*/*v*) formic acid adjusted to pH 9.0 with ammonia [19]. Sugar nucleotides were detected using negative mode electrospray ionisation tandem mass spectrometry (ESI-MS/MS). The identity of all nucleotide diphosphate sugar (NDP-sugar) species was confirmed by comparison with authentic standards (see Figure 2).

UDP-α-d-Glc is clearly the most abundant sugar nucleotide in these cells, consistent with its role in the biosynthesis of the major storage polysaccharide paramylon and its central role as a biosynthetic precursor to other sugar nucleotides [28]. There is a much smaller amount of ADP-α-d-Glc, which is used in the biosynthesis of α-glucans, such as starch and glycogen, in other organisms. These glycans are absent from Euglena, but this sugar nucleotide may be used in the biosynthesis of trehalose [29].

As mentioned above, Euglena is known to make *N*-glycans in a similar manner to other Eukaryotes. The biosynthesis of the conserved Man5GlcNAc2 core requires the transfer of GlcNAc, Man and Glc from UDP-α-d-GlcNAc, GDP-α-d-Man and UDP-α-d-Glc respectively, all of which can be detected in the cell extracts. These glycans can be further elaborated by the addition of further GlcNAc and Gal residues, with the latter coming from the UDP-α-d-Gal, which is present in quite high concentrations in these cells. However, these modifications were not detected in the present protein-bound glycan analysis (vide infra).

The surface of Euglena is known to contain glucose, galactose, mannose, fucose, xylose, and rhamnose [5] and the corresponding sugar nucleotides are present for all of these. Indeed, both UDP-β-l-Rha and trace amounts of TDP-β-l-Rha were detected, confirmed against authentic standards by LCMS. Although TDP-α-d-Glc is a precursor of the latter nucleotide, the detected high TDP-α-d-Glc concentrations indicate that it may be used for the synthesis of alternative carbohydrates, or it may be used interchangeably with UDP-α-d-Glc. The presence of UDP-β-l-Arap and UDP-α-d-GlcA at similar concentrations to GDP-β-l-Fuc and UDP-α-d-Xyl indicate there may also be capacity to synthesise significant quantities of complex glycans in Euglena.

The other major use for sugar nucleotides is in the biosynthesis of the antioxidant ascorbate, for which there are three different pathways in Euglena [4]. UDP-α-d-Glc, UDP-α-d-GlcA and GDP-α-d-Man could all be detected, but UDP-α-d-GalA and GDP-β-l-Gal were not found, despite being compared to authentic standards. The other notable sugar nucleotide detected was ADP-d-Rib, which may be involved ADP ribosylation of proteins [30] or as part of the NAD recycling pathway [31]. 

### 3.3. Glycan Analysis of Euglena

#### 3.3.1. Surface Glycans

**Figure 3:**
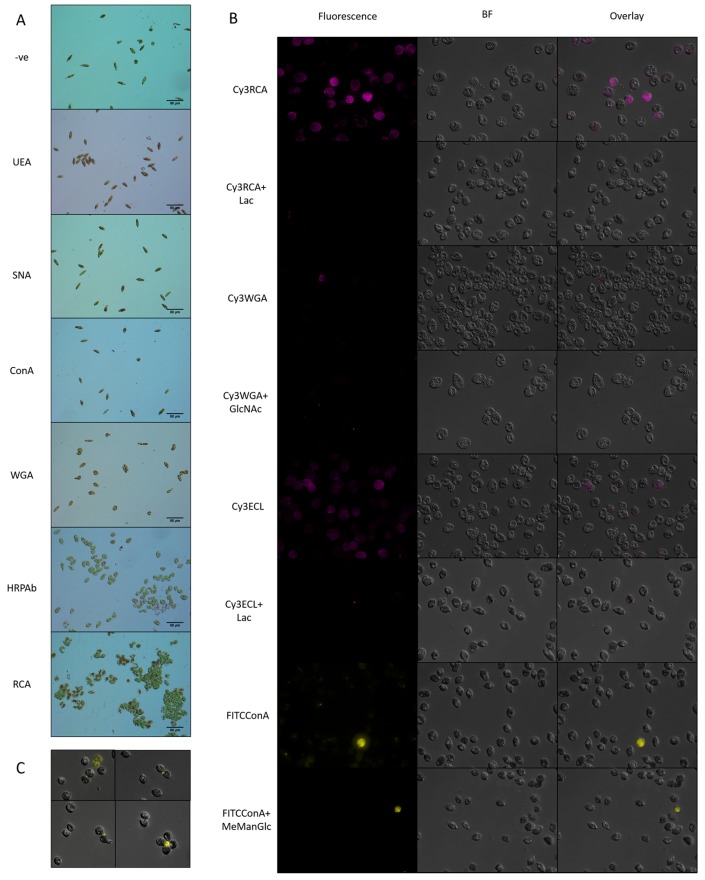
Labelling of Euglena cells with lectins. (**A**). Cells of Euglena were mixed with a variety of lectins and allowed to precipitate. They were then imaged using bright field microscopy; (**B**). Cells of Euglena were labelled with various fluorescently labelled lectins and imaged using bright field microscopy (BF) and fluorescence microscopy at the appropriate wavelength; (**C**). Representative images of dividing Euglena cells labelled with FITC-ConA.

In order to investigate the carbohydrates present on the surface of Euglena, lectins were used as probes. Initially, a panel of unlabelled lectins was incubated with Euglena and cell aggregation was monitored by eye and then by light microscopy (see Figure 3A). It was clear from this that the *Ricinus communis* agglutinin (RCA) and an antibody raised against horseradish peroxidase (HRPAb) show reactivity. These agents recognise galactose or *N*-acetylgalactosamine (GalNAc) and fucose or xylose, respectively. The absence of aggregation with Concanavalin A (ConA), *Sambucus nigra* agglutinin (SNA) or Wheat Germ Agglutinin (WGA) indicates that there is insufficient glucose- or mannose-, sialic acid- or GlcNAc-bearing oligosaccharide accessible on the surface to cross-link the cells. The small amount of crosslinking from *Ulex europaeus* agglutinin (UEA) indicates that there may be some fucose displayed on the surface of Euglena cells. 

In order to further probe Euglena cell surface glycans, fluorescently labelled lectins were employed in the presence or absence of competing ligands (see Figure 3B). The strong labelling with RCA and *Erythrina cristagalli* lectin (ECL) indicated that there are likely to be either GalNAc or Gal and Gal-β-1,4-GlcNAc moieties on the cell surface. Consistent with these observations, weak labelling with WGA indicated that there may be some GlcNAc present. ConA labelling was only apparent in cells that were disrupted and lysed, with the lectin probably labelling the paramylon storage carbohydrate (ConA binds β-glucans, but with a 28 fold lower affinity than for α-glucans [32]). Additionally, some cells in the last stages of mitosis appeared to have some labelling adjacent to the point of cell separation or at the base of the flagella (see Figure 3C), indicating that there may be some α-Glc or α-Man residues accessible at the fission point.

#### 3.3.2. Immunocarbohydrate Microarray Profiling

**Figure 4:**
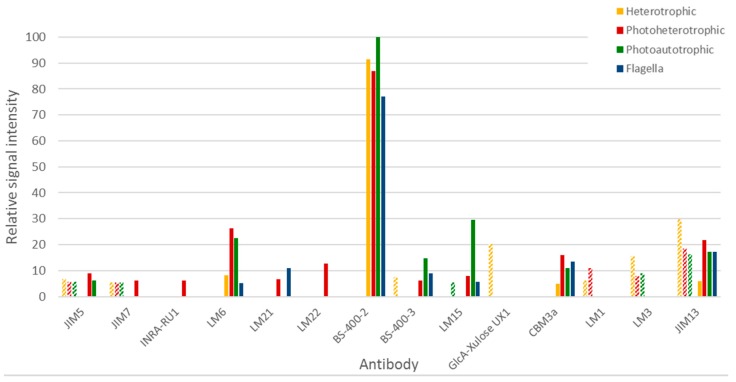
Immunocarbohydrate microarray profiling of Euglena samples. Only those antibodies that had some reactivity to the Euglena samples are presented and a full list of the antibodies used and their specificity are presented in Table S3, Supplementary Materials. Hashed bars represent CDTA released material and solid bars represent NaOH released material. Signal is normalised to the strongest signal.

Immunocarbohydrate microarray profiling is a semi-quantitative high-throughput method whereby cell wall polysaccharide components are extracted and then printed as microarrays onto membranes and probed with panels of monoclonal antibodies and/or carbohydrate-binding modules (CBMs) [23]. This provides information about the relative abundance of polysaccharide epitopes and is useful for obtaining a comparative overview of glycans across sample sets. Euglena cells grown under three different conditions (photoautotrophic, photoheterotrophic and heterotrophic) were analysed, as well as flagella isolated from heterotrophically grown cells. Two extraction solvents were used sequentially, first 1,2-diaminocyclohexanetetraacetic acid (CDTA) and then NaOH. As these extraction methods are optimised for their use on land plant cell wall materials, the data requires careful interpretation (see Figure 4).

From the milder CDTA treatment, it is apparent that no carbohydrates were released from the flagella material. CDTA treatment of the cells released material contained glycan epitopes recognised by anti-homogalacturonan, anti-extensin and anti-AGP antibodies (mAbs JIM5 and JIM7, LM1 and LM3, and JIM13 respectively). Heterotrophic cells also released material with a relatively high abundance of epitopes recognised by anti-glucuronoxylan and anti-mixed linkage glucan mAbs (mAb GlcA-Xylose UX1 and BS-400-3 respectively), and photoheterotrophic cells released material containing the xyloglucan epitope recognised by mAb LM15 (see Figure 4). The material released by NaOH had a strikingly different profile to that released by CDTA. The most notable signal was that obtained from the anti-β-1,3-glucan mAb BS-400-2 and this binding was most likely due to the presence of the major storage polysaccharide, paramylon. In addition, NaOH also released material containing epitopes recognised by cellulose binding CBM3a, and the anti-arabinan and anti-arabinogalactan mAbs LM6 and JIM13 respectively. It is also worth noting that distinctly different profiles were obtained for photoheterotrophic and photoautotrophic cells. For example, in NaOH released material, photoheterotrophic cells contained more mannan (recognised by mAbs LM21 and LM22), rhamnogalacturonan (recognised by mAbs INRA-RU1) and methylesterfied homogalacturonan (recognised by mAb JIM7) than photoautotrophic cells. In contrast, photoautotrophic cells contained relatively higher levels of xyloglucan (recognised by mAb LM15) and mixed linkage glucan (recognised by mAb BS-400-3). Taken together, these results suggest that the major carbohydrate in Euglena is paramylon, but other more structurally complex polymers related to xylan, mannan, arabinan and arabinogalactan are also present.

#### 3.3.3. Analysis of Euglena Protein Bound Glycans

**Figure 5:**
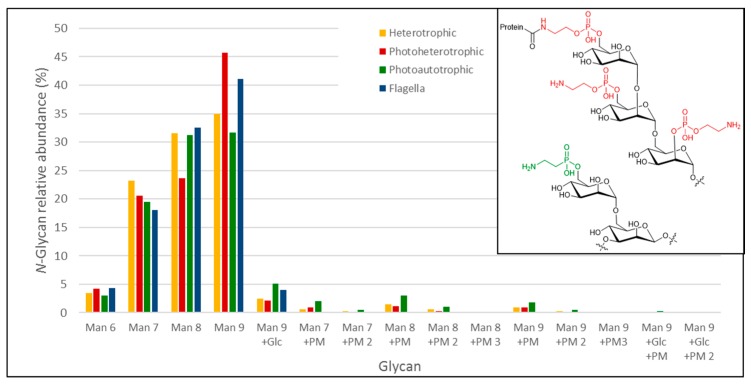
*N*-glycans found on Euglena (glyco)protein extracts and on purified flagella. For each measurement, the obtained values are normalised to the sum of all detected *N*-glycans. PM-glycan modification. The inset shows a fragment of the structure of a human GPI anchor core (upper) [38] and a fragment of a *Locusta migratoria* *N*-glycan (lower) [33]. The aminoethylphosphate and aminoethylphosphonate are highlighted in red and green respectively.

We used a porous graphitized carbon (PGC)-LC ESI MS/MS approach to identify the *N*- and *O*-glycans from Euglena photoheterotrophic, heterotrophic and photoautotrophic cells and isolated flagella. Proteins were dot-blotted onto PVDF membranes to enzymatically release *N*-glycans by PNGase F, followed by a reductive β-elimination step to release any *O*-glycans present [25]. After identification, relative quantitative data was obtained by integrating the area under the curve (AUC) obtained for the respective Extracted Ion Chromatogram (EIC’s) for each individual identified glycan compound. The *N*-glycan analysis of all of the Euglena samples revealed the exclusive presence of oligomannose-type *N*-glycans (Man9 > Man8 > Man7 > Man6 > Man9Glc) with no additional carbohydrate moieties (Figure 5 and Table 1). However, the glycomics analyses of whole protein extracts obtained from photoheterotrophic, heterotrophic and photoautotrophic *Euglena* showed that a small fraction of oligomannose-type *N*-glycans also exhibited an additional modification of 107 Da (Figure 5). Based on the detected mass differences, this 107 Da addition could reflect the attachment of a putative 2-aminoethylphosphonate to the non-reducing end of the oligomannose *N*-glycans. 2-Aminoethylphosphonate has previously been found to be attached to *N*-glycans from insects [33], and derivatives of this compound were also described to be present on marine invertebrates *N*-glycans [34]. *N*-glycans carrying a putative aminoethylphosphonate were significantly elevated in photoautotrophic (almost 10% of released glycans) and less pronounced in heterotrophic *Euglena* (~4%). Putative aminoethylphosphonate carrying *N*-glycans could only just be detected in the flagella samples, but at levels below the level of quantitation and these signals could also not be verified by tandem MS data. We did not find any indication for complex type *N*-glycans as they have been described for other algae, such as *Chlamydomonas reinhardtii*, where *N*-glycans were identified carrying both core and antenna xylose residues and some methylation [35]. Beta-elimination after PNGase F treatment did not result in any signals consistent with any *O*- or *N*-glycan-specific signatures. This leads us to conclude that *Euglena gracilis* does not possess any PNGase F-resistant, complex type glycans, previously described in other Euglenozoa [36,37], at least not in levels detectable by the overall highly sensitive and selective approach. Overall, this comprehensive *N*-glycan profiling of Euglena shows that it produces predominantly high mannose type glycans with a small proportion containing a modification consistent with the mass of an aminoethylphosphonate.

## 4. Conclusions

*Euglena gracilis* expresses a wide range of glycosyltransferases and also produces the precursor sugar nucleotides necessary for the synthesis of some very complex glycans. Our immunocarbohydrate microarray profiling indicates that the surface of Euglena has some xylan- and arabinan-type material, bearing GalNAc and GlcNAc moieties. However, these monosaccharides appear not to be used to form complex type *N*-glycans, as only oligomannose type *N*-glycans were detected in this study. There is evidence for the addition of an unusual aminoethylphosphonate to a small proportion of these *N*-glycans; aminoethylphosphate-bearing mannose residues are more typically recognised as a core component of human GPI anchor structures. Because Euglena apparently does not produce classic complex-type *N*-glycans, it offers a platform to be used for the heterologous expression and manufacture of medicinal proteins that will exclusively carry oligomannose-type *N*-glycans. Targeted engineering of selected glycosylation pathways, such as sialylation, has been successfully achieved in plants [39]. As most important nucleotide precursors are naturally available in Euglena, a similar strategy could transform it into an attractive recombinant glycoprotein production platform. This will require more detailed work to determine whether the presence of such complex type *N*-glycans affects the growth and fermentation potential of Euglena. Overall, these data indicate that *Euglena gracilis* has potential as an alga platform for the manufacture of pharmaceutical glycoproteins.

## Figures and Tables

**Table 1:** *N*-glycans identified in Euglena by PGC-LC-ESI-MS/MS.

Observed *m*/*z*	Charge State	Calculated Mass (Da)	TheoreticalMass (Da)	Delta Mass (Da)	Glycan Identified	
698.24	2	1398.48	1398.5	0.02	(Hex)3 + (Man)3(GlcNAc)2	Man 6
779.25	2	1560.50	1560.55	0.05	(Hex)4 + (Man)3(GlcNAc)2	Man 7
860.26	2	1722.52	1722.60	0.08	(Hex)5 + (Man)3(GlcNAc)2	Man 8
941.37	2	1884.74	1884.66	−0.08	(Hex)6 + (Man)3(GlcNAc)2	Man 9
1022.32	2	2046.64	2046.71	0.07	(Hex)7 + (Man)3(GlcNAc)2	Man 9 + Glc
832.75	2	1667.50	1667.55	0.05	(107)(Hex)4 + (Man)3(GlcNAc)2	Man 7 + PM
886.25	2	1774.50	1774.55	0.05	(107)2(Hex)4 + (Man)3(GlcNAc)2	Man 7 + PM 2
913.76	2	1829.52	1829.60	0.08	(107)(Hex)5 + (Man)3(GlcNAc)2	Man 8 + PM
967.26	2	1936.52	1936.60	0.08	(107)2(Hex)5 + (Man)3(GlcNAc)2	Man 8 + PM 2
1020.76	2	2043.52	2043.60	0.08	(107)3(Hex)5 + (Man)3(GlcNAc)2	Man 8 + PM 3
994.87	2	1991.74	1991.66	−0.08	(107)(Hex)6 + (Man)3(GlcNAc)2	Man 9 + PM
1048.37	2	2098.74	2098.66	−0.08	(107)2(Hex)6 + (Man)3(GlcNAc)2	Man 9 + PM 2
1101.87	2	2205.74	2205.66	−0.08	(107)3(Hex)6 + (Man)3(GlcNAc)2	Man 9 + PM 3
1075.82	2	2153.64	2153.71	0.07	(107)(Hex)7 + (Man)3(GlcNAc)2	Man 9 + Glc + PM
1129.32	2	2260.64	2260.71	0.07	(107)2(Hex)7 + (Man)3(GlcNAc)2	Man 9 + Glc + PM 2

Hex—Hexose; HexNAc—*N*-Acetyl Hexosamine; Man—Mannose; GlcNAc—*N*-acetyl Glucosamine; PM—glycan modification (107 Da) putatively assigned as 2-aminoethylphosphonate, previously described for *Locusta migratoria* apolipophorin III protein [33].

## Supplementary Materials

The following are available online at www.mdpi.com/2079-7737/6/4/45/s1, Table S1: Number of Euglena transcripts encoding proteins in each of the CAZyme families, Table S2: Standards of sugar nucleotides detected in the photoautotrophic culture of *Euglena gracilis*: relative retention times and MRM transitions, Table S3: Specificity of the antibodies used in the immunocarbohydrate microarray profiling
