# Supplementary material for: Exploring the Glycans of Euglena gracilis
Source: Biology (Basel). 2017 Dec 15;6(4):45. doi: 10.3390/biology6040045 (PMC5745450; doi:10.3390/biology6040045)
Supplement: Supplementary file 1 [file biology-06-00045-s001.pdf]

Table S1: Number of Euglena transcripts encoding proteins in each of the CAZyme families. [4] 40 transcripts encoded proteins only distantly related to characterised GT families and may represent members of new GT families. These are included in the totals for generating Figure 1.

CAZyme family	Number of Euglena transcripts	CAZyme family	Number of Euglena transcripts	Distantly related to CAZyme family	Number of Euglena transcripts	Accessory module family	Number of Euglena transcripts
GT1	15	GH1	2	GT4	1	CBM48	1
GT2	23	GH2	6	GT23	2	CBM57	1
GT4	21	GH3	12	GT25	2	CBM63-EXPN	1
GT8	17	GH5	12	GT32	8	**CBM Total**	**3**
GT10	14	GH17	3	GT34	8	CE3	1
GT13	1	GH18	4	GT47	1	CE13	8
GT15	3	GH19	1	GT61	14		
GT17	4	GH20	3	GT69	1		
GT20	1	GH27	1	GT75	1		
GT22	6	GH30	13	GT90	2		
GT23	12	GH31	8	**Distant GT Total**	**40**		
GT24	1	GH36	6				
GT25	2	GH38	4				
GT28	1	GH42	1				
GT31	13	GH43	3				
GT32	11	GH47	11				
GT33	1	GH55	1				
GT34	1	GH63	2				
GT40	2	GH64	1				
GT41	3	GH65	5				
GT47	15	GH78	1				
GT48	4	GH81	18				
GT49	2	GH85	4				
GT50	1	GH88	1				
GT57	2	GH99	1				
GT58	1	GH117	3				
GT59	1	**GHTotal**	**127**				
GT61	16						
GT66	3						
GT69	2						
GT75	2						
GT76	2						
GT77	25						
**GTTotal**	**228**						

Table S2: Standards of sugar nucleotides detected in the phototrophic culture of *Euglena gracilis*: relative retention times and MRM transitions.

Sugar Nucleotide	Relative Retention time	MRM transitions	Fragment
UDP-α-D-Glc	1.00	565 → 323<br>565 → 79	[NMP-H]$^-$<br>$[H_3PO_4\text{-}H_3O]^-$
UDP-α-D-Gal	0.92	565 → 323<br>565 → 159	[NMP-H]$^-$<br>$[H_4P_2O_7\text{-}H_3O]^-$
UDP-α-D-GlcNAc	0.98	606 → 385<br>606 → 159	$[NDP\text{-}H\text{-}H_2O]^-$<br>$[H_4P_2O_7\text{-}H_3O]^-$
UDP-α-D-GlcNAcA	0.89	620 → 403<br>620 → 159	[NDP-H]$^-$<br>$[H_4P_2O_7\text{-}H_3O]^-$
UDP-α-D-GlcA	0.74	579 → 403<br>579 → 323	[NDP-H]$^-$<br>[NMP-H]$^-$
UDP-β-L-Rha	0.84	549 → 323<br>549 → 159	[NMP-H]$^-$<br>$[H_4P_2O_7\text{-}H_3O]^-$
UDP-β-L-Ara*p*	0.81	535 → 323<br>535 → 159	[NMP-H]$^-$<br>$[H_4P_2O_7\text{-}H_3O]^-$
UDP-α-D-Xyl	0.99	535 → 323<br>535 → 159	[NMP-H]$^-$<br>$[H_4P_2O_7\text{-}H_3O]^-$
TDP-α-D-Glc	1.39	563 → 321<br>563 → 241	[NMP-H]$^-$<br>$[Glc\text{-}1\text{-}P\text{-}H\text{-}H_2O]^-$
TDP-β-L-Rha	1.35	547 → 321<br>547 → 225	[NMP-H]$^-$<br>$c[Rha\text{-}1\text{-}P\text{-}H\text{-}H_2O]^-$
GDP-α-D-Man	1.43	604 → 442<br>604 → 424	[NDP-H]$^-$<br>$[NDP\text{-}H\text{-}H_2O]^-$
GDP-β-L-Fuc	1.60	588 → 442<br>588 → 344	[NDP-H]$^-$<br>$[NMP\text{-}H\text{-}H_2O]^-$
ADP-α-D-Glc	1.65	588 → 346<br>588 → 241	[NMP-H]$^-$<br>$c[Glc\text{-}1\text{-}P\text{-}H\text{-}H_2O]^-$
ADP-D-Rib<br>[5''-(adenosine 5'-pyrophosphoryl)-D-ribose]	1.64	558 → 346<br>558 → 159	[NMP-H]$^-$<br>$[H_4P_2O_7\text{-}H_3O]^-$

Table S3: Specificity of the antibodies used in the immunocarbohydrate microarray profiling

Antibody	Specificity
JIM5	Homoglacturonan (HG) with a low DE (mAb JIM5)
JIM7	HG with a high DE (mAb JIM7)
LM18	HG Partially methylesterified (mAb LM18)
LM19	HG Partially methylesterified (mAb LM19)
LM20	HG Partially methylesterified (mAb LM20)
LM7	HG Partially methylesterified (mAb LM7)
INRA-RU1	Backbone of rhamnogalacturonan I (mAb INRA-RU1)
INRA-RU2	Backbone of rhamnogalacturonan I (mAb INRA-RU2)
LM5	(1→4)-β-D-galactan (mAb LM5)
LM9	Feruloylated (1→4)-β-D-galactan (mAb LM9)
LM6	(1→5)-α-L-arabinan (mAb LM6)
LM13	Linearised (1→5)-α-L-arabinan (mAb LM13)
LM16	Processed (1→5)-α-L-arabinan (mAb LM16)
LM12	Feruloylate on any polymer (mAb LM12)
BS-400-4	(1→4)-β-D-(galacto)mannan (mAb BS-400-4)
LM21	(1→4)-β-D-(galacto)(gluco)mannan (mAb LM21)
LM22	(1→4)-β-D-(gluco)mannan (mAb LM22)
BS-400-2	(1→3)-β-D-glucan (mAb BS-400-2)
BS-400-3	(1→3)(1→4)-β-D-glucan (mAb BS-400-3)
LM15	Xyloglucan (XXXG motif) (mAb LM15)
LM24	Xyloglucan (mAb LM24)
LM10	(1→4)-β-D-xylan (mAb LM10)
LM11	(1→4)-β-D-xylan/arabinoxylan (mAb LM11)
LM23	(1→4)-β-D-xylan (mAb LM23)
GXAb-UX1	Glucoronoxylan (mAb Anti-GlcA-Xylose UX1)
CBM3a	Celulose (crystalline) (mAb CBM3a)
LM1	Extensin (mAb LM1)
LM3	Extensin (mAb LM3)
JIM11	Extensin (mAb JIM11)
JIM12	Extensin (mAb JIM12)
JIM19	Extensin (mAb JIM19)
JIM20	Extensin (mAb JIM20)
JIM4	AGP (mAb JIM4)
JIM13	AGP (mAb JIM13)
LM14	AGP (mAb LM14)
LM2	AGP, β-linked GlcA (mAb LM2)
